# Modulation of Amyloidogenesis Controlled by the C-Terminal Domain of Islet Amyloid Polypeptide Shows New Functions on Hepatocyte Cholesterol Metabolism

**DOI:** 10.3389/fendo.2018.00331

**Published:** 2018-06-25

**Authors:** Angel Pulido-Capiz, Raúl Díaz-Molina, Israel Martínez-Navarro, Lizbeth A. Guevara-Olaya, Enrique Casanueva-Pérez, Jaime Mas-Oliva, Ignacio A. Rivero, Victor García-González

**Affiliations:** ^1^Departamento de Bioquímica, Facultad de Medicina Mexicali, Universidad Autónoma de Baja California, Mexicali, Mexico; ^2^Facultad de Enfermería, Universidad Autónoma de Baja California, Mexico City, Mexico; ^3^Instituto de Fisiología Celular, Universidad Nacional Autónoma de México, Ciudad de México, Mexico; ^4^Centro de Graduados e Investigación en Química, Instituto Tecnológico de Tijuana, Tijuana, Mexico

**Keywords:** IAPP, amyloids, cholesterol, hepatocytes, metabolism, diabetes mellitus type 2, biomimetics, insulin

## Abstract

The islet amyloid polypeptide (IAPP) or amylin maintains a key role in metabolism. This 37-residues-peptide could form pancreatic amyloids, which are a characteristic feature of diabetes mellitus type 2. However, some species do not form amyloid fibril structures. By employing a biomimetic approach, we generated an extensive panel of optimized sequences of IAPP, which could drastically reduce aggregation propensity. A structural and cellular characterization analysis was performed on the C-terminal domain with the highest aggregation propensity. This allowed the observation of an aggregative phenomenon dependent of the lipid environment. Evaluation of the new F_23_R variant demonstrated inhibition of β-sheet structure and, therefore, amyloid formation on the native C-terminal, phenomenon that was associated with functional optimization in calcium and cholesterol management coupled with the optimization of insulin secretion by beta cells. When F_23_R variant was evaluated in microglia cells, a model of amyloidosis, cytotoxic conditions were not registered. In addition, it was found that C-terminal sequences of IAPP could modulate cholesterol metabolism in hepatocytes through regulation of SREBP-2, apoA-1, ABCA1, and LDLR, mechanism that may represent a new function of IAPP on the metabolism of cholesterol, increasing the LDL endocytosis in hepatocytes. Optimized sequences with only one residue modification in the C-terminal core aggregation could diminish β-sheet formation and represent a novel strategy adaptable to other pharmacological targets. Our data suggest a new IAPP function associated with rearrangements on metabolism of cholesterol in hepatocytes.

## Introduction

Exacerbated perturbation of protein homeostasis (proteostasis) could lead to a phenomenon considered as protein metastasis (1, 2). Initial events of misfolding and amyloid aggregation trigger a cascade of pathological processes that could mark the progression of chronic-degenerative diseases (3, 4), in association with conditions of metabolic overload due to the excess of nutrients. These conditions are important in diseases such as diabetes mellitus type 2 (DM2), a pathological condition associated with reduced quality of insulin synthesis, impaired insulin sensitivity in peripheral tissues, and the presence of amyloid-like fibrils composed of human amylin (hIAPP) that affect β-cell function.

hIAPP is a peptide hormone of 37 residues that slows down gastric emptying, participates in the homeostatic regulation of glucose, and functions as a glucagon-release inhibitor and a sensitizer of leptin function (5, 6). In addition, data generated by our group suggest a regulatory function of IAPP in hepatic cholesterol metabolism. hIAPP is a monomeric peptide processed in the Golgi apparatus and co-secreted with insulin in response to β-cell secretagogues (7). Amyloid aggregation of hIAPP triggers an amplified toxicity response that can lead to failure of pancreatic β-cells. In this case, extracellular hIAPP amyloids could induce endoplasmic reticulum (ER) stress. In the lumen, ER stress is detected by IRE1, PERK, and ATF6α, a tripartite signaling system denominated the unfolded protein response (UPR). The UPR regulates gene expression to resolve the ER stress and maintain proteostasis by the signaling of ER-nucleus cascades through factors such as N-ATF6α, XBP1s, and CHOP (8). The overactivation of the UPR can promote β-cell dysfunction (9).

Different variants are involved in alterations of amyloid structures *in vivo*. For instance, the S_20_G mutation has been found in Asian populations and associated with early cases of DM2 accelerating the formation of amyloid structures (10, 11). Also several variants have been reported in Maori populations of New Zealand (12). Moreover, rat IAPP (rIAPP) shows a diminished trend to produce amyloid fibrils, the differences among human and rat sequences are six residues (H_18_R, F_23_L, A_25_P, I_26_V, S_28_P, and S_29_P), all located in segments 18–29 (13). Several reports suggest that the 23–37 domain may be key in the structural transitions that trigger the formation of amyloid fibrils (13, 14).

A nature-based approach, i.e., biomimetics associated with the adaptation of models inspired by biology, has been the basis for the generation of new systems or materials (15). The tendency to form IAPP-amyloid structures of hundreds of organisms has not been completely analyzed. A characterization of IAPP sequences could provide the basis for a better understanding of the aggregation phenomenon and the cellular response mechanisms to IAPP. Therefore, the development of improved sequences might serve as a source for optimized pharmacological treatments that may reduce the formation of β-sheet structures.

## Materials and Methods

### Analysis of Sequences

Employing the term islet amyloid polypeptide (IAPP) in the searching protein database of the NCBI, 314 sequences were found, which correspond to 284 eukaryotes and another 30 associated sequences (16). In this approach and through different filters, we considered only 240 of the 284 that integrate the characterized, predicted, and partial sequences. Using several specialized platforms, algorithms-predictors of beta-sheet aggregation and amyloid fibril formation, we described the correlation that presents hIAPP sequence with respect to the other organisms.

### Physicochemical Parameters

Physicochemical analysis of IAPP sequences was performed, included hydrophobic moment (μH), net electrostatic charge, average hydrophobicity, theoretical average rate of aggregation, isoelectric point (pI), and patterns including residues promoting membrane interactions, which have been described modulate phenomena such as self-assembling and misfolded aggregation (17, 18). Additionally, the management of IAPP fragments through the N-domain ^1^KCNTATCATQRLANFLV^17^ and C-domain ^17^VHSSNNFGAILSSTNVGSNTY^37^ was performed.

### Characterization of Aggregation Propensity

We employed several algorithms that enable the identification of regions with a high intrinsic propensity to aggregation. IAPP sequences were characterized through multiple alignments by the use of specialized platforms such as Aggrescan (19), PASTA 2.0 (20), and Zyaggregator (21, 22).

### Phylogenetic Tree Construction

Sequence similarity search was realized with the method “Basic Local Alignment Search Tool protein” (BLASTp), which performs protein–protein sequence comparison and employ heuristic model (20, 23). In addition, other strategies were used to reconstruct the phylogenetic tree as the NJ method and ClustalX software (24, 25).

### Sequences Designed *De Novo*

Based on network analysis, different residues among species were replaced on the sequence of IAPP. Then, the effect of substitution was characterized through physicochemical and aggregative parameters. Aggregation propensity range was obtained by consider the aggregation value of hIAPP as a reference, this based in Aggrescan values (Na4vSS).

### Materials

Cell culture reagents were purchased from Gibco-Invitrogen, while tissue culture plates and other plastic materials were obtained from Corning. Salts and buffers were obtained from Sigma-Aldrich, as well as thioflavin T (ThT), black Sudan B, Congo-red, SDS, [3-(4, 5-dimethylthiazol-2-yl)-2, 5-diphenyltetrazolium bromide] tetrazolium (MTT), tunicamycin (Tum), and lipopolysaccharides O111:B4 (LPS). l-α-phosphatidylcholine (PC), 1-oleoyl-2-hydroxy-sn-glycero-3-phosphate (LPA) were obtained from Avanti Polar Lipids.

### Peptide Synthesis and Preparation

Several peptides were synthesized (GenScript) considering the amino acid sequence of IAPP and physicochemical properties. Specifically, the following sequences were characterized: ^1^KCNTATCATQRLANFLVHSS^20^ (N-terminal native); ^23^FGAILSSTNVGSNTY^37^ (C-terminal native), a region described with a high tendency to aggregation, and ^23^RGAILSSTNVGSNTY^37^ (F_23_R variant). The best condition for peptide solubilization was ultrapure H_2_O (0.6 mg/mL), subsequently diluted to phosphate buffer pH 7.2 (0.06 mg/mL). Peptide purity greater than 98% was confirmed by mass spectrometry and HPLC analysis (GenScript). Likewise, the aggregative core of amyloid beta (Aβ) was used as a control ^25^GSNKGAIIGLM^35^. All solutions were filtered by membranes with a pore size of 0.22 µm.

### Lipid Vesicles

The lipid effect on peptide structure was performed using ThT-fluorescence, peptide bond spectroscopy, and biochemical techniques. Lipids were mixed in chloroform and dried for 1 h under a gentle stream of N_2_ with an additional incubation of 5 h at 30°C in a Speed Vac concentrator, according to protocols established by our working group (4). After drying, lipids were resuspended in a phosphate buffer and subsequently sonicated with four cycles of 10 min (15 s on/30 s off pulses) in an ice bath under a N_2_ flow. Samples were incubated 2 h at room temperature and then centrifuged for 10 min at 13,000 rpm. Solution of small unilamellar vesicles obtained was kept at 4°C until use, previously characterized by transmission electron microscopy (18).

### Peptide-Bond Conformational Changes

Experimentation was performed through characterization of absorbance at 218 nm, associated with changes in the conformation of the peptide-bond, a parameter of conformational changes in the formation of beta-sheet structures (4, 18). Measurements were obtained using a BioRad Smart spectrophotometer with diode array.

### Congo-Red Birefringence Spectroscopy

Assays were performed based on the protocol of Ref. (26) and adapted by our group in several papers. Employing 10.6 µM of Congo-red and 60 µg/mL of peptides under the different conditions evaluated, the optical density was measured every 2 nm from 400 to 700 nm.

### ThT Fluorescence

Beta-sheet structures in peptides were characterized with ThT fluorescence assay. Fluorescence emission spectra were registered at 25°C from 460 to 610 nm with an excitation wavelength of 450 nm in a Cary Eclipse Fluorescence spectrophotometer.

### Circular Dichroism (CD)

Circular dichroism spectra were recorded with an AVIV 62DS spectropolarimeter (AVIV Instruments) at 25°C employing far UV wavelengths (190–260 nm). Experiments were conducted at a peptide concentration of 120 µg/mL in a 1 mm quartz path length cuvette using AVIV software. Spectra were recorded with a 1 mm bandwidth, using 1 nm increments and 2.5 s accumulation time. CD results were reported as mean molar ellipticity (deg cm^2^ dmol^−1^).

### Electronic Microscopy Experimentation

Peptide samples incubated under the different conditions tested (2 µL) were deposited on Formvar-Carbon TEM grids, copper 300 mesh; and after incubation for 2 min at 25°C, excess liquid was removed with Whatman paper. Peptide concentrations were adjusted to 60 µg/mL in 20 µM phosphates buffer pH 7.4. Images were acquired using a JEOL-7800F Prime Field Emission Scanning electron microscopy with scanning transmission electron microcopy detector at 30.0 kV.

### Lipid–Peptide Interactions

Lipid/peptide samples were analyzed on non-denaturing electrophoresis, technique adapted by our group in lipid–peptide characterization (27). We established a new methodology through the use of 0.8–15% native gradient gel electrophoresis. Later, gradient gels were stained with Sudan-black and silver protocols, according to previous work (28).

### Cell Culture

RIN-m5F beta cells (Langerhans islets, ATCC) were cultured in RPMI 1640 medium supplemented with l-glutamine and 10% fetal bovine serum (FBS). Likewise, microglia cells (EOC) were maintained in DMEM medium supplemented with 10% FBS and 20% LADMAC conditioned media (produced from the LADMAC cell line, rich in growth factor colony stimulating factor 1). In a complementary way, we used hepatocytes (C9 cells) in DMEM medium with FBS 10%. Penicillin (50 U/mL) and streptomycin (50 g/mL) were added to the media.

### Cell Viability Assay

Peptide cytotoxicity was assessed by MTT reduction assays in cell cultures, under different peptide and lipid–peptide treatments. Cells were seeded into 96-well plates at a density of 20,000 cells/well and allowed to grow to 90% of confluence. Next, culture medium was replaced with Opti-MEM medium. After 1 h under this condition, cells were incubated under the different treatments, and subsequently processed according to previous protocols (4).

### Preparation of Lipopolysaccharides (LPS)

LPS O111:B4 were diluted in ultrapure H_2_O at a concentration of 1 × 10^6^ ng/mL sonicated for 10 min, filtered by 0.22 µm, and subsequently diluted in culture medium at different concentrations.

### Preparation of Fatty Acids (FA)

Palmitic acid (PA) and oleic acid (OA) were prepared in an ethanol/H_2_O solution (1:1; vol:vol) at 60°C to reach a final concentration of 75 mM. Subsequently, PA and OA were incubated with free fatty acid-bovine serum albumin (BSA) for 2 h at 37°C, filtered by 0.22 µm, and then diluted in culture medium under different concentrations with a final molar ratio of 4:1 (FA/BSA), before adding the treatment to the culture plates.

### Western Blot

For the evaluation of cholesterol metabolism and UPR pathway, cell cultures were maintained in proliferation until 90% of confluence, then, cells were incubated under different treatments for 16 and 24 h. Cells were washed with PBS and lysed for 35 min at 4°C with protein buffer lysis supplemented with protease and phosphatase inhibitors. Further to centrifugation at 8,500 rpm for 8 min, the supernatant was recovered and the protein quantification was carried out using the bicinchoninic acid procedure. Samples (20 µg/lane) from the total protein fraction were analyzed by SDS-PAGE on 10% gels, further transferred to PVDF membranes (Millipore). Membranes were incubated 1 h at 37°C in a solution containing TBS, 0.1% tween-20, and 5% non-fat milk (blocking solution). For protein detection, the following primary antibodies were used: anti-PMCA-ATPase, anti-CHOP, anti-IL-6, anti-LDLR, anti-SREBP2, anti-ABCA1, anti-apoA1, anti-N-ATF6α, anti-XBPIs, and anti β-actin as a loading control. Blocked membranes were incubated with the primary antibodies overnight at 4°C. After washing, horseradish peroxidase (HRP)-conjugated secondary antibodies were incubated with the membrane for 1 h at 37°C in blocking solution. The secondary antibodies used were: donkey anti-goat IgG, goat anti-rabbit IgG, and goat anti-mouse IgG. Later, membranes were washed with TBS/0.1% tween and HRP activity detected with the Immobilon Western kit (Millipore).

### Determination of Insulin

Supernatant media of RIN-m5F cells under different treatments were recovered and then centrifuged at 5,000 rpm for 2 min. Recovered supernatants were diluted with PBS. Subsequently, employing the ALPCO insulin quantification kit (80-INSHUU-E01) with a sensitivity of 0.002 ng/mL, the insulin determination was performed as specified by manufacturer.

### LDL Fraction

Human plasma sample was obtained from a healthy donor in the Facultad de Medicina Mexicali, which signed an informed consent. All protocols were performed according to the Declaration of Helsinki. To isolate LDL, plasma density was adjusted to a 1.019–1.063 g/mL by adding KBr and then centrifuged at 360,000 *g* for 8 h at 4°C. The layer containing VLDL and IDL was discarded. LDL fraction was recovered and dialyzed in 150 mM NaCl solution, filtered through 0.45 µm, and maintained under a nitrogen atmosphere at 4°C to reduce oxidation. Native LDL concentration was measured with the bicinchoninic acid method. Labeling of LDL with 1,1′-dioctadecyl-3,3,3′3′-tetramethylindocarbocyanine-perchlorate (DilC18) (Molecular Probes) was performed by incubation of probe 15 µg for 1 mg of LDL-protein, 8 h at 25°C (dil-LDL). Then, solution was adjusted to a density solution of 1.053 g/mL and centrifuged 90,000 rpm for 3 h. The dil-LDL fraction was recovered and dialyzed to PBS. Protein determination was performed and dil-LDL was employed in hepatocyte experimentation (29).

### Cell Cytometer Assays

Prior to the internalization experiments, hepatocyte cultures at 90% confluence were incubated in FBS-free medium. After 1 h of fasting, cells were treated with different fragments of IAPP, then, cells were incubated with dil-LDL (3 µg/mL) for 24 h. Cellular characterization was performed in a Beckman-Coulter cytometer, 20,000 events were registered. To measure fluorescence of dil-LDL fluorescence, a PC5.5-a filter was employed.

## Results

### Importance of Regions 17–31 in IAPP Sequences

**Figure 1:**
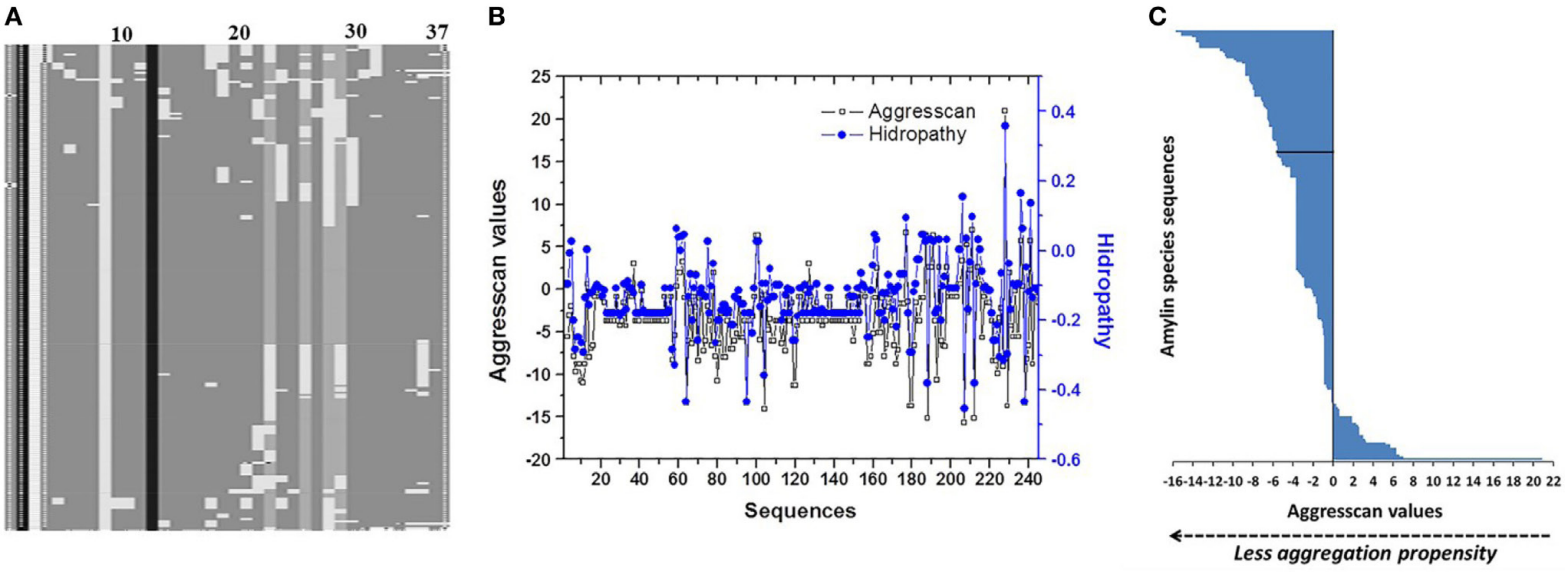
Analysis of primary structure of islet amyloid polypeptide (IAPP) among 240 species. **(A)** Evaluation of multiple alignments of 240 sequences showing the conserved sites (24). **(B)** Analysis of correlation among Aggrescan and hydropathy values of sequences. **(C)** Distribution of Aggrescan values throughout 240 sequences of IAPP. Negative values were associated with lower aggregation propensity, and positive values with higher aggregation propensity. In black line is identified the sequence of hIAPP.

A panel of 240 IAPP sequences was selected according to the criteria described in Section “Materials and Methods,” the most important groups were distributed by class such as mammalia (97 sequences), aves (103), actinopterygii (25), and sauropsida (12). Specifically, aves class was distributed more evenly among 36 different orders, followed by mammalia composed of two main groups, primates and rodentia (Table S1 in Supplementary Material). Alignments in 240 IAPP sequences suggests that the N-terminal domain (1–17 residues) was conserved in almost all sequences, showing minimal changes (Figure 1A), highly conserved residues were C_2_, L_12_, and L_16_. However, some positions showed the highest number of variants among species such as residue S_29_ with 211 sequences, H_18_ with 209, F_23_ with 206, and A_8_ with 168 (Table 1). The regions 17–31 concentrated 74% of total variants (1,187 of 1,640), showing the greatest degree of variability. Employing the bioinformatics platform BLASTp, a high correlation was found among 49 sequences aligned in the N-terminal (residues 1–17 of hIAPP) with at least 88% of identity, compared to the C-domain (residues 23–37), which was only found in 11 sequences with 90% of identity, corresponding to primates. Additionally, based on phylogenetic tree analysis and multiple alignments among N- and C-domains in 240 sequences, the N-domain was conserved among a wide variety of organisms. On the other hand, C-domain was restricted to phylogenetically close groups (Figure S1 in Supplementary Material). The C-terminal domain of hIAPP could show major structural modifications and possibly a domain where conformational changes trigger beta-sheet formation. Specifically, regions 23–31 represent the site where protein frustration takes place, indicating that it could be the key for the binding of ligands (30, 31). Thus, residue modifications in this region could reduce protein frustration, originating a stable core of protein folding and slowing down interactions among monomers.

**Table 1:** Primary structure of hIAPP placed in order position, showing the different variants that were originated from the comparison among 240 sequences, the frequency of each variant is shown.

Position	Amino acid	Variants								Residue variants	Total of sequences with the specific variants
1	K	K_1_R	K_1_E	K_1_I						3	10
2	C									0	0
3	N	N_3_G	N_3_D							2	15
4	T	T_4_I	T_4_M							2	6
5	A	A_5_V	A_5_I	A_5_P						3	7
6	T	T_6_I								1	1
7	C	C_7_Y								1	1
8	A	A_8_V	A_8_S	A_8_E	A_8_T					4	168
9	T	T_9_M	T_9_I							2	12
10	Q	Q_10_H								1	5
11	R	R_11_E	R_11_W	R_11_H						3	5
12	L									0	0
13	A	A_13_T	A_13_V	A_13_S						3	16
14	N	N_14_D	N_14_T	N_14_H						3	153
15	F	F_15_Y								1	1
16	L									0	0
17	V	V_17_D	V_17_G	V_17_T	V_17_A	V_17_I	V_17_S			6	34
18	H	H_18_R								1	209
19	S	S_19_T	S_19_F	S_19_A						3	4
20	S	S_20_G	S_20_N	S_20_C	S_20_R					4	28
21	N	N_21_S	N_21_G	N_21_H						3	43
22	N	N_22_S	N_22_T	N_22_I	N_22_K	N_22_G				5	66
23	F	F_23_I	F_23_L	F_23_M	F_23_R	F_23_A	F_23_V	F_23_G		7	206
24	G	G_24_R	G_24_A	G_24_S						3	4
25	A	A_25_T	A_25_D	A_25_P	A_25_V	A_25_S	A_25_I			6	52
26	I	I_26_V	I_26_A	I_26_L	I_26_M	I_26_Y				5	74
27	L	L_27_Y	L_27_I	L_27_F	L_27_S	L_27_H	L_27_X			6	166
28	S	S_28_V	S_28_A	S_28_L	S_28_P	S_28_T				5	53
29	S	S_29_P	S_29_A	S_29_T	S_29_H	S_29_L				5	211
30	T	T_30_V	T_30_P	T_30_R	T_30_N					4	5
31	N	N_31_K	N_31_D	N_31_S	N_31_V					4	32
32	V	V_32_M	V_32_L	V_32_G						3	10
33	G	G_33_S								1	1
34	S	S_34_A	S_34_P							2	4
35	N	N_35_S	N_35_A	N_35_T	N_35_Y	N_35_D	N_35_G	N_35_H	N_35_K	8	30
36	T	T_36_A	T_36_S							2	5
37	Y	Y_37_H								1	3

*The amino acids of the hIAPP sequence are shown in gray shades*.

### Characterization of the Propensity to Amyloid Fibril Formation

**Figure 2:**
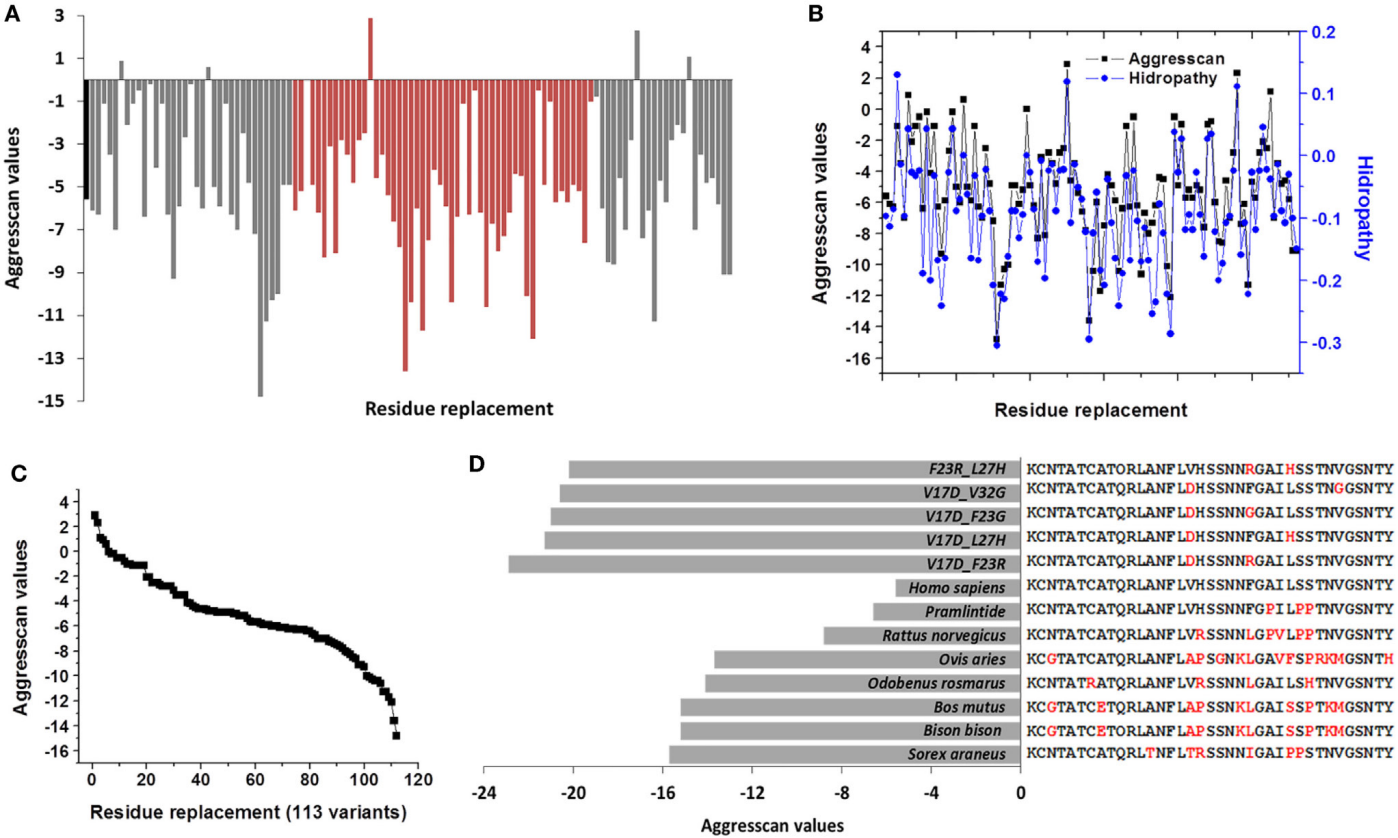
Single modifications on hIAPP could originate 113 *de novo* sequences that modulate the amyloid aggregation in islet amyloid polypeptide. **(A)** Effect of residue substitution on the hIAPP (residues 1–37), showed by the Aggrescan values. Orange variants in regions 17–31 are identified; black bar corresponds to hIAPP. **(B)** Association of hydropathy and Aggrescan values of 113 *de novo* variants. **(C)** Sigmoidal trend was registered in aggregation values of 113 *de novo* variants. **(D)** Sequences of species with low aggregation tendency in contrast to less aggregative sequences *de novo* with two substitutions. hIAPP was used as a reference, in red, were identified the variants.

Analysis of hydropathy values regarding the aggregation propensity index obtained with the Aggrescan algorithm (Na4vSS value) identified a correlation among the values of these variables (Pearson 0.82) (Figure 1B). This correlation was not displayed when values of parameters such as μH, average hydrophobicity, or isoelectric point, were evaluated. Based on the Aggrescan values, sequences such as *Sorex araneus* (−15.7 Na4vSS), *Bos mutus* (−15.2), *Bison bison* (−15.2), *Odobenus rosmarus* (−14.1), and *Ovis aries* (−13.7) showed the lowest aggregation values (Table S2 in Supplementary Material). The analysis of 240 sequences showed 61 with low aggregation propensity and 173 with a higher trend compared to hIAPP. Indeed, hIAPP is situated in a midpoint among sequences (Figure 1C). With the use of algorithms such as PASTA 2.0 and Zyggagregator, similar results were obtained. Importantly, through the evaluation of sequences, we found 113 different residue variants in comparison to hIAPP (Table 1). In regions 17–31 of IAPP, 60% of the substitutions were concentrated, and the replacement of each of the 113 variants in their respective position on the hIAPP sequence was characterized (Figure 2A).

Using as a reference the value of hIAPP (−5.6), 43% of sequences decreased the tendency to aggregate, which could modify the nucleation phenomenon in IAPP monomers. Similarly, when Aggrescan and hydropathy values were evaluated together, a correlation value of 0.87 (Pearson) was found (Figure 2B); in other physicochemical parameters, there was not a significant association. Aggrescan analysis in 113 new sequences suggests a sigmoidal behavior (Figure 2C), where most of the sequences could be situated in potential intermediate transition states of aggregation. For instance, in 15 sequences with less aggregation, variants such as V_17_D, F_23_R, F_23_G, and I_26_A could delete any of the *hot spot*s (Table 2). In general, when positions 17, 23, 25, 26, and 32 in hIAPP were modified, the tendency to aggregation was reduced.

**Table 2:** Effect of substitutions of individual variants on human amylin sequence.

Variant	Sequence	Na4VSS	Hidroph.	PASTA	Organisms
V_17_D	KCNTATCATQRLANFL**D**HSSNNFGAILSSTNVGSNTY	−14.8	−0.31	−3.46	*S. crofa*
F_23_R	KCNTATCATQRLANFLVHSSNN**R**GAILSSTNVGSNTY	−13.6	−0.30	−3.88	*C. auratus*
L_27_H	KCNTATCATQRLANFLVHSSNNFGAI**H**SSTNVGSNTY	−12.1	−0.29	−4.10	*L. chalumnae*
F_23_G	KCNTATCATQRLANFLVHSSNN**G**GAILSSTNVGSNTY	−11.7	−0.18	−3.88	*P. cristatus*
V_32_G	KCNTATCATQRLANFLVHSSNNFGAILSSTN**G**GSNTY	−11.3	−0.22	−4.21	*P. cristatus*
V_17_G	KCNTATCATQRLANFL**G**HSSNNFGAILSSTNVGSNTY	−11.3	−0.22	−3.46	*A. nancymaae, B. regulorum, S. boliviensis*
I_26_A	KCNTATCATQRLANFLVHSSNNFGA**A**LSSTNVGSNTY	−10.6	−0.17	−3.88	*C. cristata, C. porcellus, F. damarensis*
A_25_D	KCNTATCATQRLANFLVHSSNNFG**D**ILSSTNVGSNTY	−10.4	−0.24	−3.95	*C. cristata*
F_23_A	KCNTATCATQRLANFLVHSSNN**A**GAILSSTNVGSNTY	−10.4	−0.12	−3.88	*G. fortis, Z. albicollis,*
V_17_T	KCNTATCATQRLANFL**T**HSSNNFGAILSSTNVGSNTY	−10.3	−0.23	−3.46	*C. harengus, E. lucius, S. araneus*
L_27_S	KCNTATCATQRLANFLVHSSNNFGAI**V**SSTNVGSNTY	−10.1	−0.22	−6.19	*B. bison, B. mutus, C. cristata, P. cristatus*
V_17_A	KCNTATCATQRLANFL**A**HSSNNFGAILSSTNVGSNTY	−10.0	−0.16	−3.46	*B. bison, B. mutus, B. taurus, B. bubalis*
A_8_E	KCNTATC**E**TQRLANFLVHSSNNFGAILSSTNVGSNTY	−9.3	−0.24	−4.94	*B. bison, B. mutus, B. taurus*
Y_37_H	KCNTATCATQRLANFLVHSSNNFGAILSSTNVGSNT**H**	−9.1	−0.15	−4.94	*C. hircus, O. aries*
T_36_S	KCNTATCATQRLANFLVHSSNNFGAILSSTNVGSN**S**Y	−9.1	−0.10	−4.94	*O. princeps, O. cuniculus*
*H. sapiens*	**KCNTATCATQRLANFLVHSSNNFGAILSSTNVGSNTY**	−5.6	−**0.10**	−4.94	
K_1_I	**I**CNTATCATQRLANFLVHSSNNFGAILSSTNVGSNTY	−1.1	0.13	−4.94	*P. hamadryas,*
S_29_L	KCNTATCATQRLANFLVHSSNNFGAILS**L**TNVGSNTY	−1.0	0.03	−6.36	*P. alecto lq*
S_28_L	KCNTATCATQRLANFLVHSSNNFGAIL**L**STNVGSNTY	−1.0	0.03	−6.36	*C. porcellus, F. damarensis, H. glaber*
T_30_V	KCNTATCATQRLANFLVHSSNNFGAILSS**V**NVGSNTY	−0.8	0.04	−6.86	*O. prínceps*
S_28_V	KCNTATCATQRLANFLVHSSNNFGAIL**V**STNVGSNTY	−0.5	0.04	−7.61	*A. limnaeus, C. variegatus, F. heteroclitus*
A_25_I	KCNTATCATQRLANFLVHSSNNFG**I**ILSSTNVGSNTY	−0.5	−0.02	−7.41	*P. cristatus*
A_5_I	KCNT**I**TCATQRLANFLVHSSNNFGAILSSTNVGSNTY	−0.5	−0.02	−4.94	*O. prínceps*
T_9_I	KCNTATCA**I**QRLANFLVHSSNNFGAILSSTNVGSNTY	−0.2	0.04	−4.94	*C. cristata, F. damarensis, H. glaber*
T_6_I	KCNTA**I**CATQRLANFLVHSSNNFGAILSSTNVGSNTY	−0.2	0.04	−4.94	*A. nancymaae*
S_19_F	KCNTATCATQRLANFLVH**F**SNNFGAILSSTNVGSNTY	0.0	0.00	−6.50	*C. cristata*
R_11_W	KCNTATCATQ**W**LANFLVHSSNNFGAILSSTNVGSNTY	0.6	0.00	−5.84	*P. alecto lq*
T_4_I	KCN**I**ATCATQRLANFLVHSSNNFGAILSSTNVGSNTY	0.9	0.04	−4.94	*A. nancymaae*
N_35_Y	KCNTATCATQRLANFLVHSSNNFGAILSSTNVGS**Y**TY	1.1	−0.04	−4.94	*L. chalumnae, P. gutturalis*
N_31_V	KCNTATCATQRLANFLVHSSNNFGAILSST**V**VGSNTY	2.3	0.11	−6.97	*P. cristatus*
N_22_I	KCNTATCATQRLANFLVHSSN**I**FGAILSSTNVGSNTY	2.9	0.12	−3.46	*P. cristatus*

*In order, 15 sequences with lower aggregation and 15 with greater aggregation are showed, compared to hIAPP. Hot spots regions are identified in red*.

*Hidroph., hydrophobicity in kilocalorie per mole*.

### Proposal of *De Novo* Sequences

Pramlintide is administered in diabetes therapeutic and considered a non-aggregating peptide based on rat IAPP (rIAPP) (32); however, this *de novo* design could not represent the best strategy. In the analysis of the sequence of pramlintide with three substitutions, a value of −6.6 (Na4vSS) was registered, and the rIAPP sequence presents −8.8. Moreover, incubation of equimolar concentrations of rIAPP and hIAPP leads to a deposition of rIAPP onto amyloid fibrils of hIAPP (33). This may have a significant relevance in the efficacy of pramlintide, suggesting the possibility of new optimized sequences. Considering the panel of 113 *de novo* variants, 30 of these show less aggregation propensity than pramlintide, with a single residue substitution on hIAPP sequence. Otherwise, taking into account these variants (Table 2) and performing the replacement of two residues on the sequence of hIAPP could generate about 95 sequences with lower aggregation index.

Compared to *Homo sapiens*, sequences such as *Sorex araneus* (Aggrescan −15.7) with 6 variants, *Bos mutus* (−15.2) with 10, and *Odobenus rasmarus* (−14.1) with 4 variants had the least tendency to aggregation (Table S2 in Supplementary Material). Contrasting significantly with *de novo* sequences with only two substitutions, such as V_17_D_F_23_R (Aggrescan −22.9), V_17_D_L_27_H (−21.3), V_17_D_F_23_G (−21), V_17_D_V_32_G (−20.6), or F_23_R_L_37_H (−20.2). Even more, variants V_17_D_F_23_R and V_17_D_F_23_G could delete *hot spots* (Figure 2D). In this sense, modifications that are close to positions 17 and 23 of IAPP showed a lower aggregation propensity. Replacements by polar residues such as Asp and Arg were the most effective to reduce aggregation. Indeed, in the evaluation of sequences with three variants in the sequence of hIAPP data contrast significantly with pramlintide and rat values, obtaining 644 new sequences less prone to aggregation (data not shown).

### Modulation of IAPP Folding

Variants that could increase the formation of beta-sheet structures were found in organisms such as *P. cristatus, L. chalumnae, A. nancymaae*, and *P. alecto*. For instance, only in the evaluation of a group of 15 selected variants, changes that induce a greater propensity to aggregation were N_22_I (2.9 Na4vSS), N_31_V (2.3), N_35_Y (1.1), T_4_I (0.9), and R_11_W (0.6), broadening *hot spots* in hIAPP (Table 2). These clusters have been associated with atomic structures of the spine of amyloid fibrils made up of beta-sheets with interdigitating side chains, denominated steric zippers (34). Variants in the regions 21–28, 24–33, and 32–37 could increase the tendency to aggregation (Table 2), and substitutions at positions 11, 21, and 28 were the most relevant to induce amyloid aggregation, as well as changes for Ile, Val, Tyr, and Trp, generating long and more hydrophobic *hot spots*.

**Figure 3:**
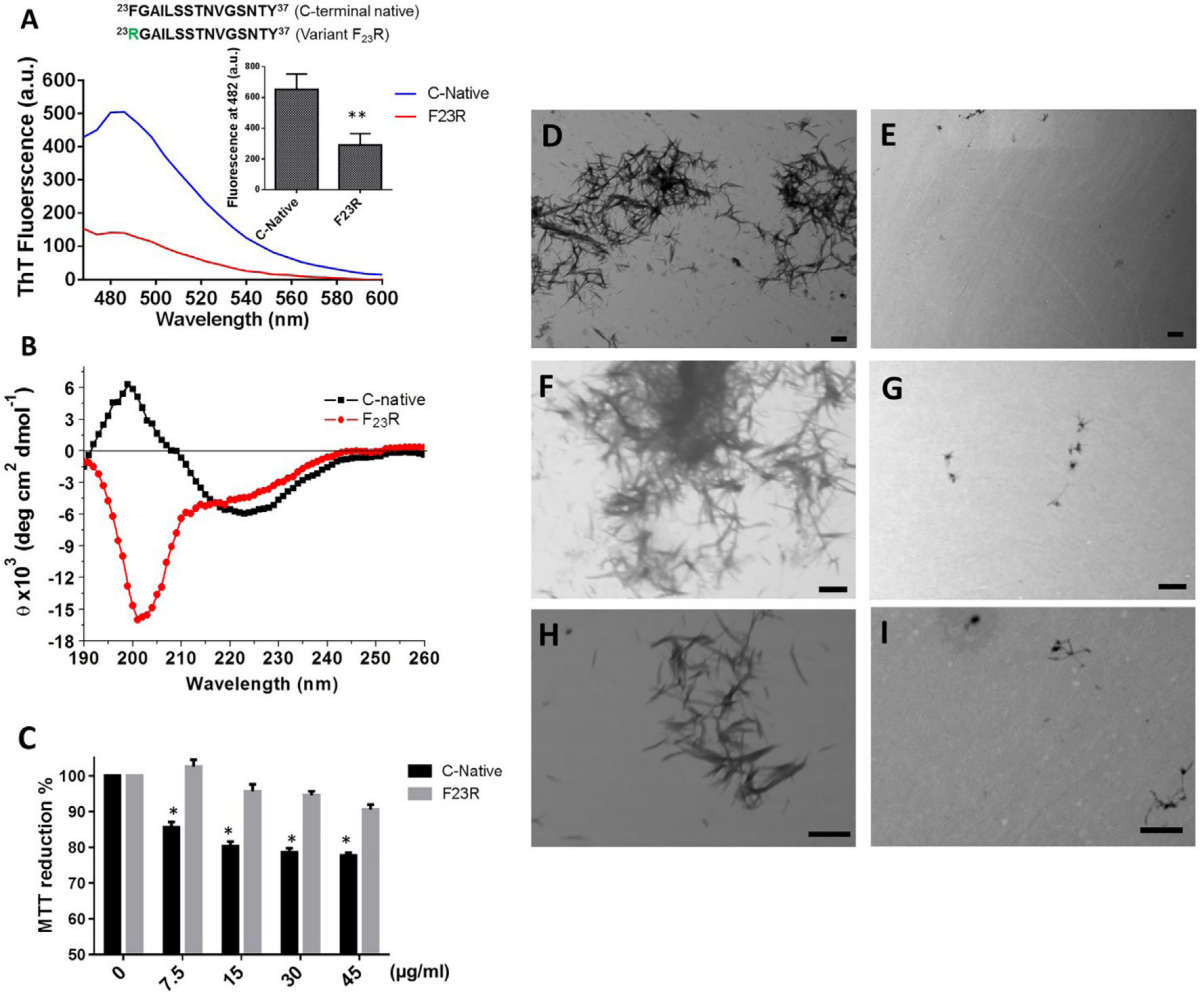
The F_23_R variant modulates beta-sheet formation in C-terminal 23–37 islet amyloid polypeptide domain. **(A)** Emission values obtained with ThT-fluorescence assay, the peak was recorded at 482 nm. **(B)** Circular dichroism spectra of the variants. **(C)** Cell viability evaluated through MTT assay in RIN-m5F cells under increasing concentrations of peptides. Mean values were presented (*n* = 6, X ± SD) *p < 0.001, **p < 0.005. Peptide samples processed by transmission electron microscopy of C-terminal **(D,F,H)** and F_23_R variant **(E,G,I)**. Bars correspond to 500 nm.

To evaluate the proposal of subtle modifications in the native C-terminal domain of hIAPP could reduce the aggregation propensity and modulate the deleterious effects of beta-sheet formation, the domain that intrinsically showed the highest aggregation tendency was characterized (residues 23–37) using the variant F_23_R as a peptide reference (Figure 3A). Considering its location in the aggregation core and that it was one of the variants that may induce a lower aggregation propensity (Table 2). Native C-terminal, intrinsically showed a high tendency to formation of beta-sheet structures with the characteristic emission ThT-fluorescence spectrum peak at 494 nm, consistent with previous reports (35). However, in the case of the F_23_R variant, there was a significant reduction in the formation of this type of structures (Figure 3A). Complementary experiments through characterization of CD confirmed these results. Characteristic peaks at 201 and 221 nm were associated with the presence of beta-sheet structures only at the native C-terminal (Figure 3B). In addition, when the fragment corresponding to the N-terminal domain (residues 1–17) was characterized, significant changes were not found during the structural evaluation through ThT-fluorescence and CD, keeping the sequence a disordered structure (data not shown).

**Figure 4:**
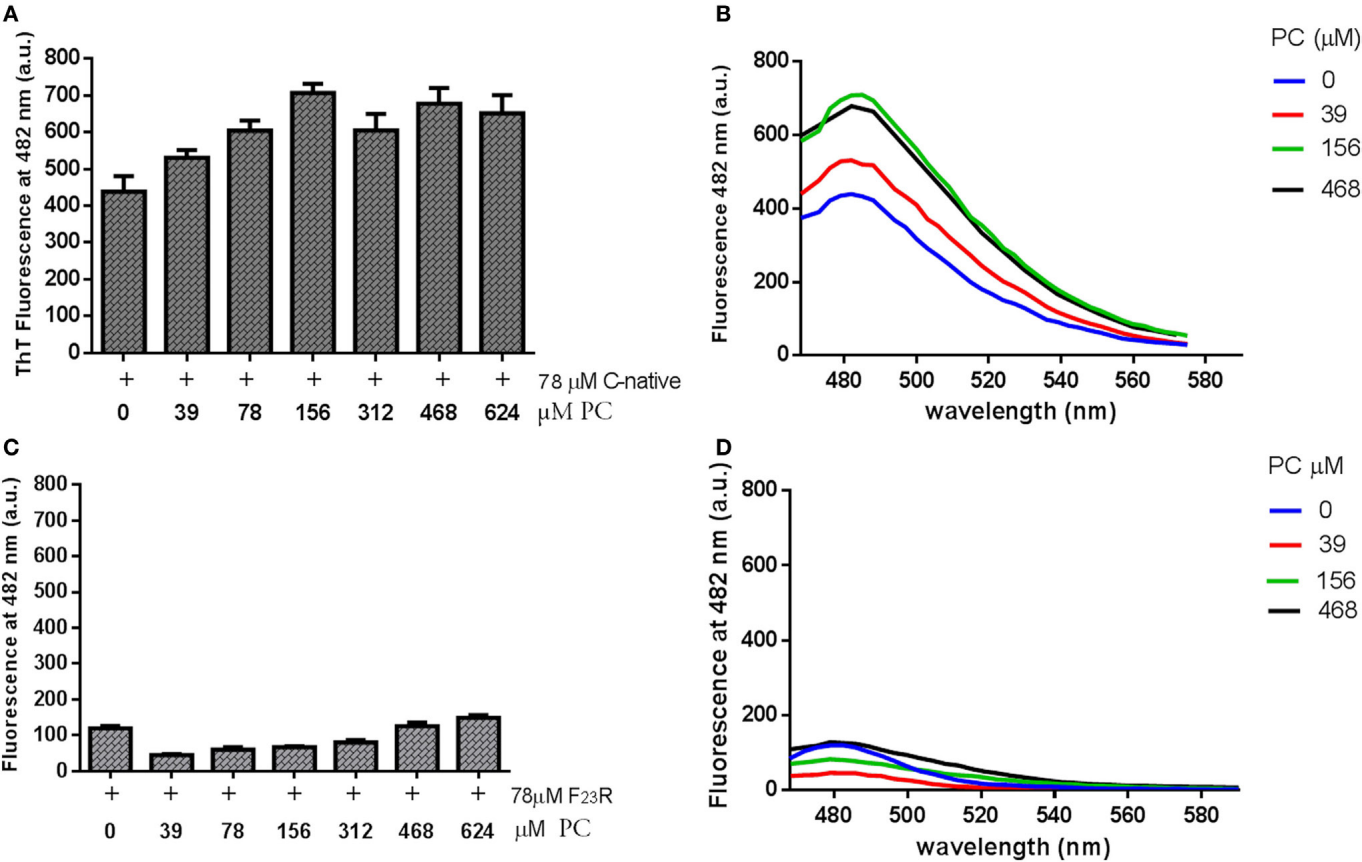
Effect of phosphatidylcholine (PC) vesicles on secondary structural changes in peptides derived from islet amyloid polypeptide. **(A)** Fluorescence values at 482 nm in the native C-terminal incubated with PC vesicles. **(B)** Under the same conditions, the emission spectra were shown. **(C)** Fluorescence values in the F_23_R variant incubated with increasing concentrations of PC. **(D)** Under the same conditions, the emission spectra were obtained.

Extracellular accumulation of hIAPP is one of the conditions that trigger the pathophysiological phenomena associated with DM2 (36), such as diminution in the cell mass of Langerhans islets. Therefore, employing a beta cell model (RIN-m5F), increasing concentrations of peptide fragments were evaluated with the MTT assay. Only the C-native fragment showed conditions of cytotoxicity since the lowest concentration evaluated (7.5 µg/mL) (Figure 3C). Considering the low percentages of β-secondary structures associated to F_23_R, minimum levels of cytotoxicity were registered. In addition, when peptide samples were processed by scanning transmission electronic microscopy, we found amyloid-like fibrils only in C-native (Figures 3D,F,H) under the same experimental conditions in F_23_R samples, the amount of amyloid fibrils was low (Figures 3E,G,I). In this sense, localized changes in secondary structure of peptides and proteins explain the activation of the function or, in some cases, misfolding protein phenomena, which could be dependent of the lipid microenvironment (4, 18). We have characterized these conditions in proteins that show lipid-binding properties and proposed that lipid–protein interactions were critical in conformational changes that lead to misfolding phenomenon (37). In this sense, a lipid microenvironment-dependence on the structure of IAPP sequences was evaluated (Figure 4).

### Effect of Lipid Environment on Secondary Structure

C-terminal and F_23_R peptides were incubated with neutral small unilamellar vesicles composed of PC. The presence of conformational transition toward the formation of beta-sheet structures was found in the native C-terminal from the lowest concentration of PC, specifically in the peptide–lipid 78/39 μM relationship (Figures 4A,B), which is close to the critical micelle concentration of PC. This phenomenon did not occur when the variant F_23_R was evaluated (Figures 4C,D). These findings may be associated with the low stability of the C-native sequence and its high propensity of aggregation. Considering that the membrane environment may enhance aggregation, and subtle changes such as the F_23_R in the domain of greater aggregation could avoid this phenomenon. Additionally, the characterization was extended to vesicles in which the electrostatic charge on the surface was modulated finding that peptide–lipid interactions that trigger amyloid formation were primarily dependent on the negative charge of lipid surfaces (personal communication).

**Figure 5:**
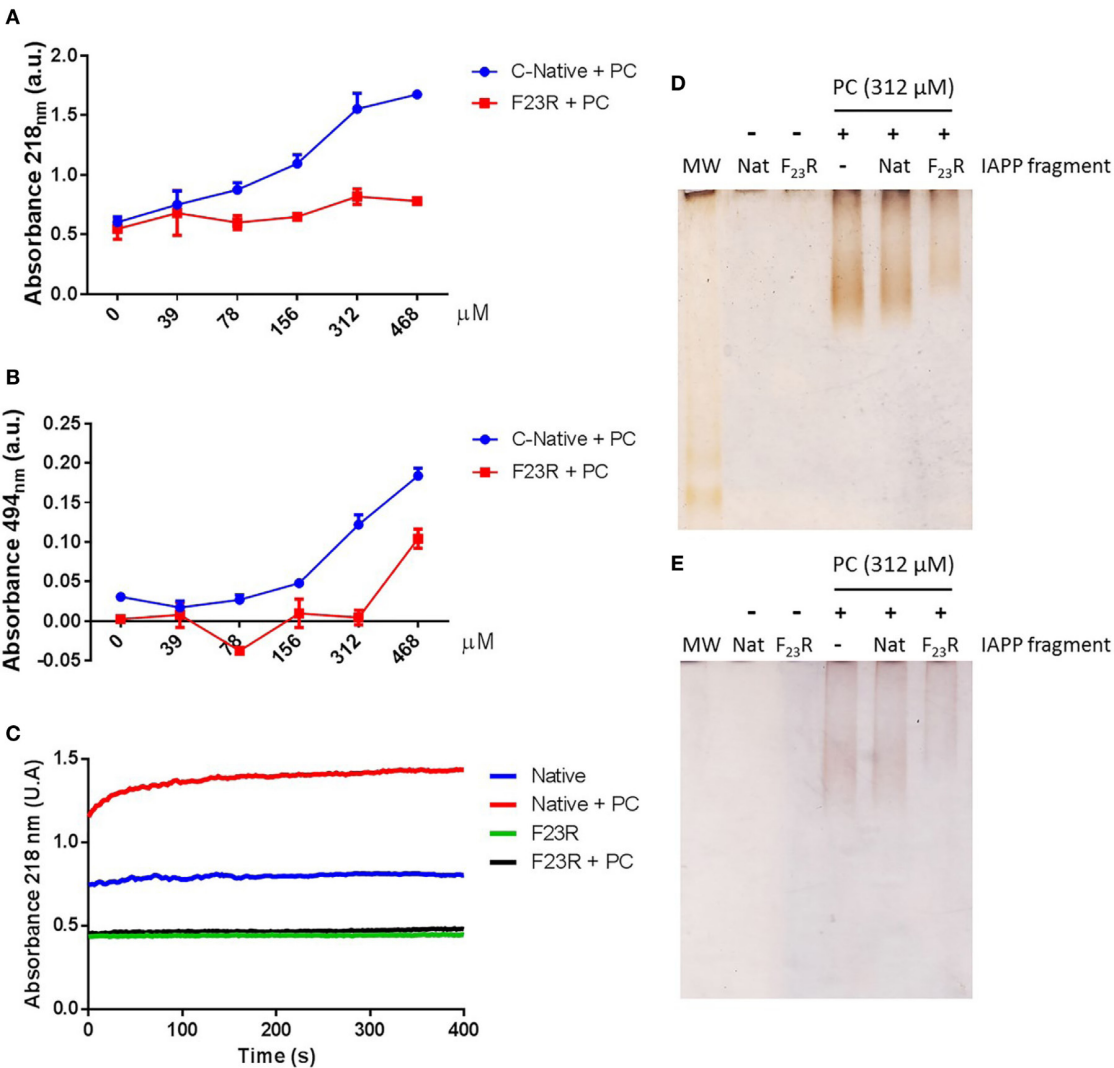
F_23_R inhibits conformational changes toward the formation of beta-structures in the C-terminal of islet amyloid polypeptide. **(A)** Evaluation of peptide-bond conformational changes in the native C-terminal and F_23_R in increasing phosphatidylcholine (PC) concentrations. **(B)** Under the same conditions, values of birefringence change by Congo-red assay. **(C)** Effect of treatment with PC on the kinetics of aggregation. Characterization of lipid/peptide interactions by native polyacrylamide gradient gels (1–15%) and processed by silver **(D)** and Sudan black **(E)** staining.

Complementarily, a dependence of the lipid environment was observed only in the native C-domain in the peptide-bond conformation (Figure 5A). Confirmed with the birefringence changes evaluated with Congo-red spectroscopy (Figure 5B) indicating the presence of beta-sheet structures dependent on PC. Likewise, this condition was confirmed by monitoring the aggregation kinetics, reflecting the same behavior in the characterization of the peptide bond, in turn, related to an increase in the content of beta-sheet structures in the native C-domain (Figure 5C). However, these changes were not observed when the F_23_R variant was evaluated. Through a standardized assay developed in our group that evaluates lipid–peptide interactions (28, 37), samples were processed through native polyacrylamide gradient gels, which evidenced the native interaction among PC vesicles with the C-terminal peptide, a condition that decreased in the F_23_R variant (Figures 5D,E).

Indeed, F_23_R and the C-terminal did not change substantially with respect to the average hydrophobicity. However, when parameters such as the Aggrescan values were considered, the native C-domain showed a value of 6.6, but the F_23_R variant was −5.3, suggesting a very low tendency to aggregation. This condition determined by electrostatic charges of side chains may explain the high aggregation propensity in C-domain. For instance, the C-terminal at pH range of 3.0–8.5 showed a net charge of 0 with an intrinsic tendency to aggregation, which has already been widely discussed in several reports as a critical problem in the therapeutics of IAPP (38). However, under this same range, the charge of the variant F_23_R was around +1. A subtle balance among the highly dynamic secondary structure of the C-terminal domain of IAPP, the net charge, and the physicochemical properties of the surrounding lipid microenvironment could define the type of secondary structure acquired.

### Inhibitory Mechanism of the F_23_R Variant on the C-Terminal hIAPP Aggregation

**Figure 6:**
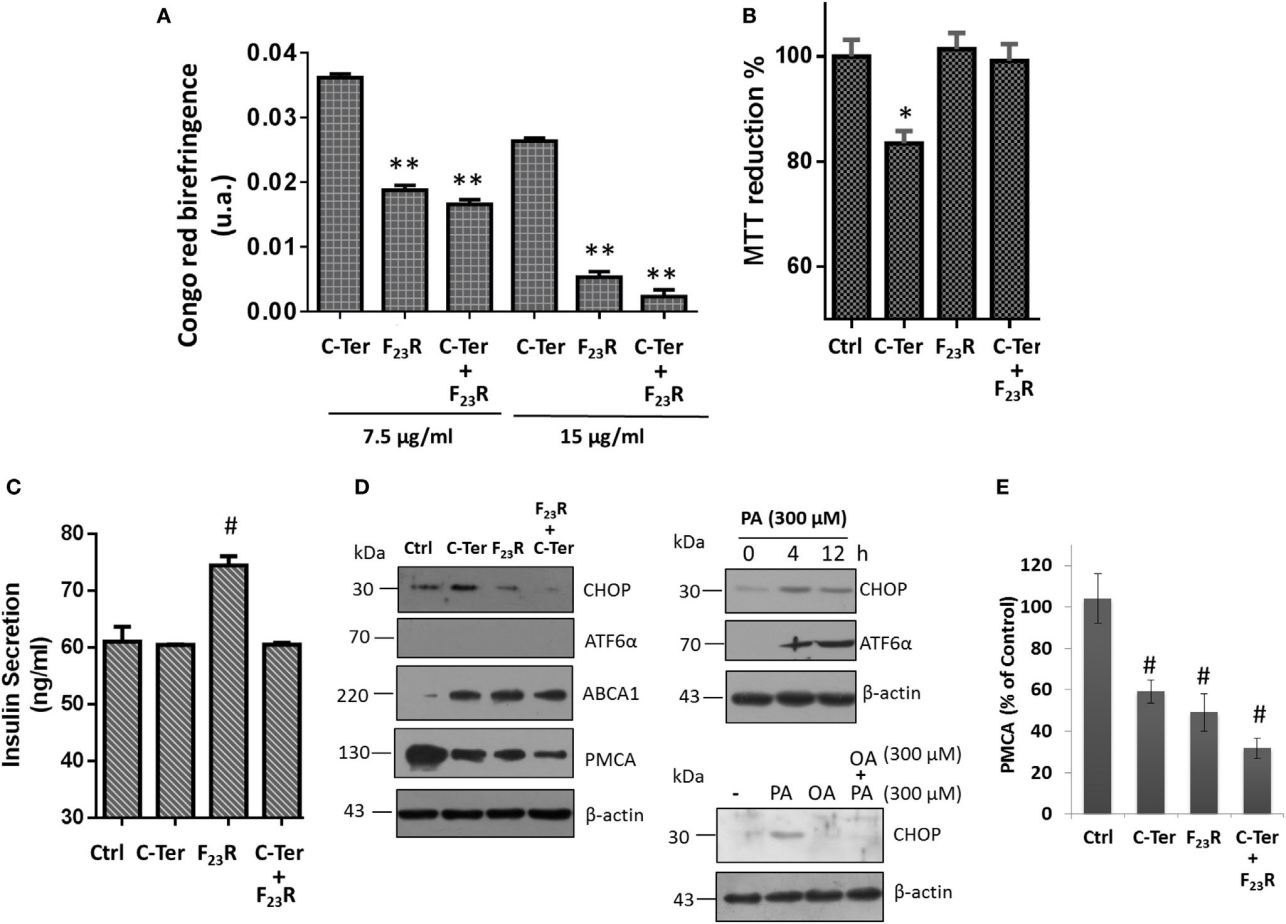
Modulation of amyloid aggregation in the native C-terminal dependent of treatment with F_23_R. **(A)** Characterization of beta-sheet structures by Congo-red birefringence assay under equimolar concentrations of peptides. **(B)** Beta-cell viability assay by MTT at peptide concentrations of 7.5 µg/mL. **(C)** Insulin quantification in extracellular media under the same conditions. Mean values are presented (*n* = 6, *X* ± SD) *p < 0.001, **p < 0.005. **(D)** Expression of CHOP and ATF6α transcription factors, as well as PMCA and ABCA1 in islet amyloid polypeptide peptide treatments. In this experiment, several controls were evaluated, effect of PA incubation on the expression of CHOP and ATF6α at 4 and 12 h. Under the same concentration (300 µM), oleic acid effect was evaluated on CHOP expression. β-actin was used as a load control. **(E)** Quantification of PMCA expression compared to control, ^#^*p* < 0.01.

Taking into consideration that the C-terminal may represent a template structure for pramlintide aggregation or other amyloid-like sequences, experimentation was performed to evaluate the interaction between the C-terminal and F_23_R under equimolar concentrations. Results suggest that the variant F_23_R can inhibit amyloid aggregation in the C-terminal, characterized by the change of birefringence with Congo-red (Figure 6A) and ThT-fluorescence (data not shown). Importantly, when cell viability was evaluated, F_23_R did not induce cytotoxic effects in beta cells and avoided cytotoxicity exerted by the native C-terminal (Figure 6B). Insulin secretion was quantified to characterize the physiological function. Although the C-terminal induced a decrease of approximately 20% in cell viability, it did not alter insulin release. Surprisingly, the F_23_R variant promoted an increase of 28% on secreted insulin levels (Figure 6C). These data suggest that F_23_R could stimulate the optimal function of beta cells. We have shown that amyloid aggregation is a phenomenon that induced the activation of UPR transducers. In this regard, treatment with the C-terminal peptide favored an increase in the expression of CHOP transcription factor dependent of PERK activation, but not ATF6α activation. An experimental control of ATF6α expression with tunicamycin was performed, an inhibitor of protein *N*-glycosylation (Figure S2A in Supplementary Material). The alteration of CHOP did not occur with F_23_R treatment or co-incubation of both peptides (Figure 6D), conditions associated with the protection phenomenon of F_23_R. Likewise, the lipotoxicity induced by the saturated palmitic acid (PA) as a positive control of the UPR activation was characterized (Figure 6D), triggering the expression of CHOP. However, employing another control condition that possibly does not induce the PERK activation of UPR, oleic acid (OA) treatment did not promote the CHOP expression; moreover, co-incubation of PA and OA avoid the ER stress (Figure 6D). Indeed, in a recently work, we have analyzed the effects main dietary FA have upon pancreatic β-cell metabolism, such as modification in protein homeostasis and intracellular management of lipid metabolism, which in turn have repercussions on insulin β-cell metabolism (39).

Although the mechanism that regulates insulin release has been described extensively through the ATP-sensitive potassium channel, other pathways can also play an important role. Indeed, beta cells express a double system for cytoplasmic Ca^2+^ extrusion: plasma membrane Ca^2+^-ATPase (PMCA) and Na^+^/Ca^2+^ exchange transport system (40). Therefore, calcium disposal is modulated by mechanisms highly regulated. In this sense, we found that the C-terminal fragment and F_23_R variant could show an association with a downregulation in PMCA expression (Figures 6D,E), connected with maintaining basal insulin-secretion levels and specifically under treatment with F_23_R allowing an optimized increase. Therefore, IAPP could exert a mechanism in the regulation of cytoplasmic calcium through the modulation of the PMCA expression, and in turn modulate the release of insulin (Figure 6C). However, PMCA overexpression has been associated with ER Ca^2+^ depletion, leading to ER-stress in beta cells (41).

The canonical apoptosis pathway due to high levels of CHOP activates the ER oxidoreductin-1α (ERO-1α) generating an increase in ER lumen hyperoxidation. Subsequently, an increase of the IP3 receptor originate a continuous release of calcium (42). Then, activation of enzymes such as calpain, which could process the proteolytic cleavage of Bcl-2 and PMCA (43). In our experiments, when this possibility was evaluated, degradation products were not registered in the PMCA characterization, suggesting that regulation is through transcriptional or translational mechanisms. In this sense, expression of PMCA could be mediated by mechanisms associated with the calcineurin/NFAT pathway (43).

In parallel, results suggested that treatment with the C-terminal and F_23_R variant modulate the cholesterol metabolism in beta cells through switching the ABCA1 transporter (Figure 6D). Modulation of the transporter can function as a compensatory mechanism that reduce cholesterol levels in cytoplasmic membrane to ameliorate the amyloid damage of IAPP, considering that cholesterol has been described as a promoter of fibril formation in hIAPP (44). Moreover, accumulation of cholesterol due to alterations in ABCA1 has been linked to impaired exocytosis of insulin granules (45). Therefore, the increase in ABCA1 could be associated with a mechanism to maintain basic functions such as insulin secretion in close connection with the regulation of PMCA expression. Taken together, this could be a novel response in beta cells to keep their basal functions.

**Figure 7:**
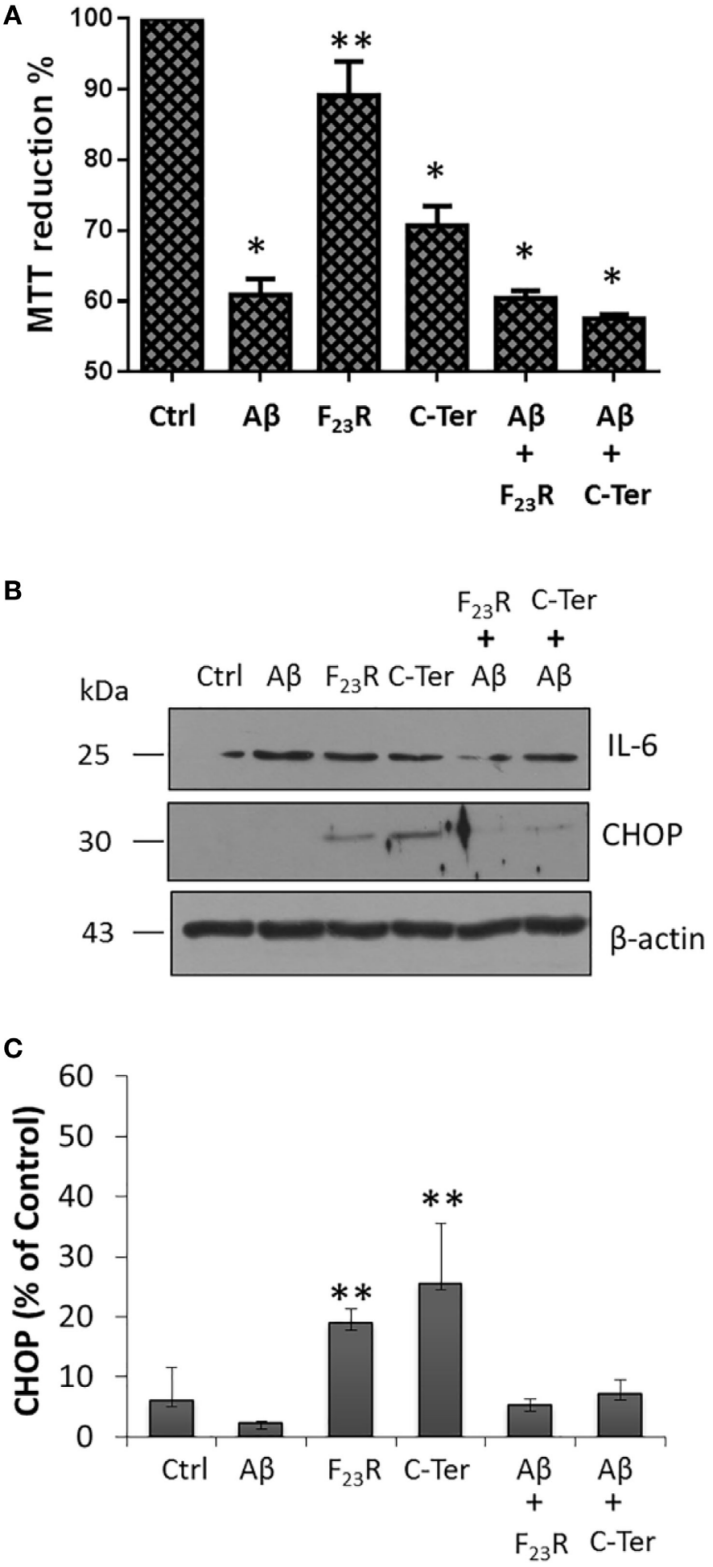
Functional modulation of islet amyloid polypeptide sequences in microglia. **(A)** Responses of cytotoxicity obtained in the microglia model, using the peptide Aβ as a reference (*n* = 6, *X* ± SD) *p < 0.001, **p < 0.05. **(B)** Under the same treatments, CHOP and IL-6 were evaluated, **(C)** quantification of CHOP expression compared to β-actin control.

Additionally, we evaluated the possibility of protection mechanisms employing the F_23_R sequence in other cellular models that are physiologically exposed to amyloid fibrils. Using Aβ-peptide as a reference amyloid, a drastic decrease in cell viability of microglia was registered, a phenomenon that was also present in C-terminal treatment (Figure 7A). Importantly, F_23_R treatment did not affect the viability of microglia. It should be noted that unlike Langerhans beta cells, equimolar treatment with F_23_R was not able to avoid damage of amyloid structures, considering that microglia is a sensitive model for proteostasis disruption. However, treatments with F_23_R induced a lower synthesis of the pro-inflammatory cytokine IL-6 (Figure 7B). CHOP is a transcription factor that regulates the expression of chaperones and redox enzymes; however, under chronic overactivation of ER stress, CHOP triggers apoptosis (2). Under our 12-h treatment in microglia, the activation of CHOP by IAPP could be associated with protective phenomena (Figures 7B,C).

### Regulation of Cholesterol Metabolism in Hepatocytes

**Figure 8:**
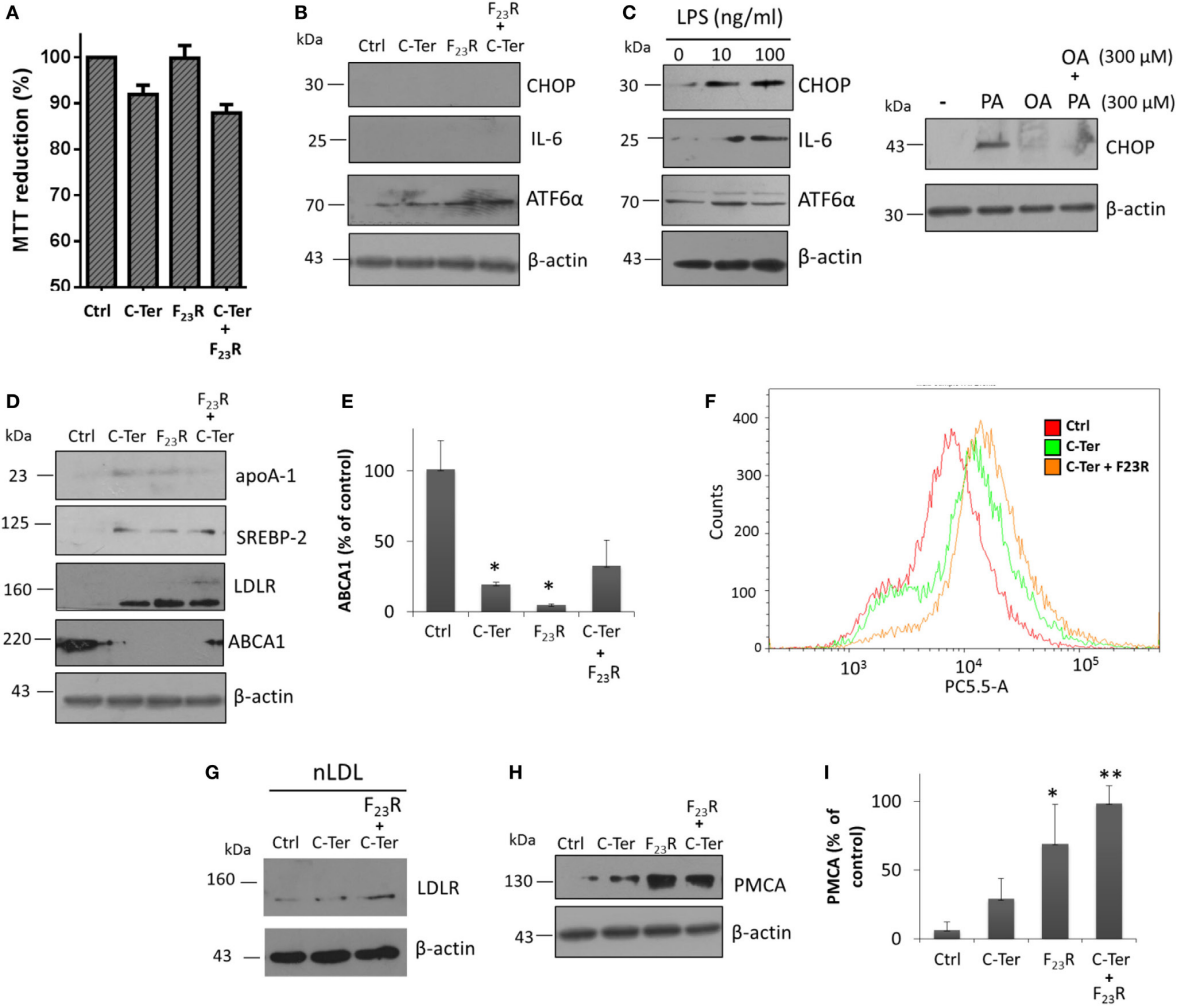
Islet amyloid polypeptide (IAPP) sequences modulate cholesterol metabolism in hepatocytes. **(A)** Cells were treated with equimolar concentrations of peptide variants (7.5 µg/mL), and MTT assay was performed, **(B)** as well as CHOP, IL-6 and ATF6α expression were evaluated. **(C)** LPS incubation was used as a positive control of CHOP and IL-6 activation. In addition, fatty acids PA and oleic acid were employed as a regulator condition of CHOP expression. **(D)** Proteins targets that regulate cholesterol metabolism were characterized, under IAPP peptide incubation. **(E)** Quantification of ABCA1 expression compared to β-actin control. **(F)** Cytometer analysis on internalization of dil-LDL (3 µg/mL). **(G)** Expression of LDLR under hepatocyte stimulation with native dil-LDL. **(H)** Western-blot of PMCA, and **(I)** quantification of PMCA expression compared to β-actin control. For western-blot analysis, *p < 0.05, **p < 0.01 compared to control.

Hepatocytes that intrinsically express low levels of CHOP were evaluated. Using the same panel of peptide treatments, no response was found in the activation of CHOP, and IL-6 synthesis was not even registered. Instead, ATF6α was activated, which was associated with minimal modification of cell viability (Figures 8A,B). Specifically, in order to show positive controls, ER stress inducer molecules were evaluated, tunicamycin and palmitic acid (Figures S2B,C in Supplementary Material), which activated in an important way the PERK pathway. Therefore, the IAPP fragments did not induce severe ER stress conditions. In addition, an inflammatory condition induced by Gram-negative endotoxins was evaluated; in this case, LPS promoted the expression of CHOP and pro-inflammatory IL-6 (Figure 8C). Also, similar to characterization of beta cells, PA-lipotoxicity induced CHOP activation; however monounsaturated OA under the same concentration did not promote this phenomenon, moreover avoided the PA effect (Figure 8C).

Characterization of several targets involved in cholesterol metabolism was performed regardless of the IAPP variants evaluated. Peptide treatment modulated cholesterol metabolism through activation of transcriptional factors that regulate expression of key targets of cholesterol metabolism such as SREBP-2. This mechanism is activated *via* COPII vesicles for the transport from ER to the Golgi apparatus similar to ATF6α, thus, there could be a shared activation mechanism. In turn, this phenomenon was associated with increased expression of the LDLR (Figure 8D). Therefore, in order to evaluate the endocytic capacity, hepatocyte cultures were stimulated with dil-LDL. Results suggested that C-terminal treatment, and F_23_R/C-terminal induced an increase of dil-LDL internalization (Figure 8F), and under these conditions confirmed by a trigger on LDLR levels (Figure 8G).

Likewise, these conditions promoted the expression of apoA-1, the major protein component of atheroprotective high-density lipoproteins. Importantly, there was a decrease in the expression of the ABCA1 transporter (Figures 8D,E). In contrast to beta cells, cholesterol maintenance in hepatocytes was associated with an increase in PMCA levels (Figures 8H,I), which induced a differential regulation of cytoplasmic calcium in connection with cholesterol metabolism. In general, these results suggest that IAPP regulates through C-terminal domain the management of hepatic cholesterol by the activation of mechanisms that favor an increase in cellular levels. Therefore, this metabolic modulation could be associated with IAPP-mediated atheroprotective functions. In this sense, one of the control mechanisms in expression of these targets may be owing to the function of long non-coding RNAs such as DYNLRB2-2:1, LeXis, or APOA1-AS (46–48), which we are currently studying.

## Discussion

Considering that peptide folding is triggered mainly by hydrophobic interactions, electrostatic properties at the polar/non-polar interface of the lipid/protein vesicles could play a key role in the transition and stabilization of IAPP segments (4). Associated with the formation of amyloid-like fibrils, specifically with the function and stability of the C-terminal domain of greater aggregation in IAPP. Otherwise, the incubation of the new F_23_R variant with the native C-terminal could modulate the conformational rearrangements and reduce the processes of amyloid fibril formation. This represents a strategic point for the improved amylinomimetic design, and, in consequence, for metabolic management.

New approaches to understand the sequences prone to aggregation and modulation of the formation of toxic-aggregate structures must be aimed at acquiring knowledge about the behavior of IAPP in different species. The generation of stable IAPP analogs may represent an optimized treatment for the regulation of glucose metabolism, and as we have documented in this paper, in the cholesterol metabolism. A biology-based strategy can provide a comprehensive solution to design-optimized sequences based on IAPP, increasing the alternative of treatments.

Alterations of cholesterol metabolism may contribute to the impairment of beta cell function (49). Our data suggest that the C-terminal and, specifically, the F_23_R variant could decrease cholesterol levels in the membrane through ABCA1. In contrast, results in the hepatocytes model suggest the presence of mechanisms that allow the accumulation of cholesterol by increased expression of the main HDL protein, apoA-1, activation of factor SREBP2, and LDLR activity. It is highly possible that tissue-specific regulation by IAPP is present. Currently, the role of IAPP in cholesterol metabolism has not been elucidated. For instance, one study conducted in rats, which subcutaneous administration of IAPP changes the proportion of trihydroxy and dihydroxycholates in the secretion of bile, improving the processes of conjugation and hydroxylation in hepatocytes (50). In addition, although it has been reported that pramlintide treatment reduced circulating triglycerides in DM2 patients, significant differences in blood pressure, HDL, and LDL cholesterol has not been found (50).

The F_23_R variant is an important proposal considering that induces a conformational rearrangement in the IAPP C-terminal reducing fibril formation processes, which is a strategic point for amylinomimetic design and, consequently, for the metabolic management. This *de novo* sequence with only one substitution could represent an optimized treatment, with a reduction in adverse effects due to immunologic activation. Although, previous studies have proposed more than one substitution of different amino acids in the N- and C-terminal domains of the native sequence of hIAPP (51). In our study evaluating beta cells, the F_23_R showed inhibitory properties of template mechanisms that trigger amyloid fibril formation in the native C-terminal domain. In turn, optimizing key functions such as insulin secretion. Importantly, in hepatocytes, F_23_R induced the metabolic rearrangement through the modulation of cholesterol flow, which could have an impact in reduction of cardiovascular risk, favoring LDL endocytosis.

Our work presents a new approach for the understanding of strategies that modulate the folding in sequences with a high propensity to aggregation through a biomimetic approach. Our data suggest that domains with a high intrinsic propensity to aggregation tend to be protected by diverse strategies to decrease this phenomenon, indicating the existence of evolutionary factors of resistance to aggregation (21). Adaptation and modulation of these phenomena is critical. Therefore, we are currently working in the chemical optimization of the structure of these peptides, which may increase their thermodynamic stability. We are currently on the tip of the iceberg about the possibilities of sequences generated *de novo* as a source of inspiration for the development of therapeutic strategies.

## Author Contributions

Conceived and designed the experiments: AP-C, IM-N, and VG-G. Performed experiments: AP-C, IM-N, LG-O, EC-P, and VG-G. Analyzed data: RD-M, JM-O, IR, AP-C, IM-N, and VG-G. Contributed reagents/materials/analysis tools: VG-G. Wrote the paper: AP-C, IM-N, and VG-G.

## Conflict of Interest Statement

The authors declare that the research was conducted in the absence of any commercial or financial relationships that could be construed as a potential conflict of interest.
