# Supplementary material for: Modulation of Amyloidogenesis Controlled by the C-Terminal Domain of Islet Amyloid Polypeptide Shows New Functions on Hepatocyte Cholesterol Metabolism
Source: Front Endocrinol (Lausanne). 2018 Jun 25;9:331. doi: 10.3389/fendo.2018.00331 (PMC6026639; doi:10.3389/fendo.2018.00331)

**Supplementary Materials**

**Supplementary figure 1.** Representation of the phylogenetic tree of the amylin sequences analyzed, based on multiple alignments among N- and C- domains, employing BLASTp algorithm. The bar is a scale for the length of branch, which represents the change among sequences of species.


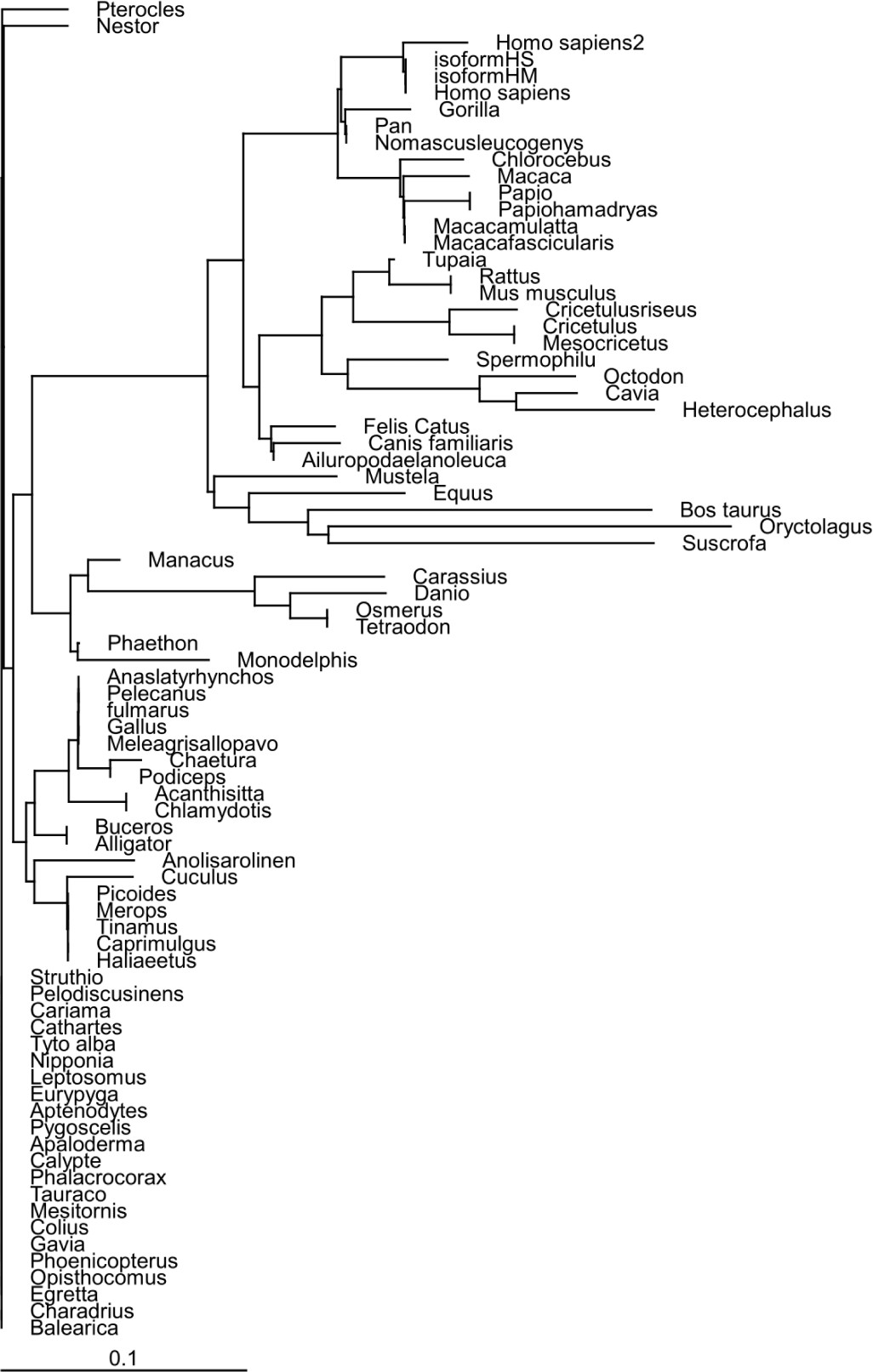


**Supplementary table 1.** Classification of 240 amylin sequences

| **Class** | **Mammalia** |  | **Chordata** |  | **Actinopterygii** |  | **Aves** |  | **Sauropsida** | **Sarcopterygii** | |  |
| --- | --- | --- | --- | --- | --- | --- | --- | --- | --- | --- | --- | --- |
| Order | *Afrosoricida* | 2 | *Beloniformes* | 1 | *Characiformes* | 3 | *Accipitriformes* | 4 | *Crocodilia* | 3 | *Coelacanthiformes* | 2 |
|  | *Artiodactyla* | 11 |  |  | *Clupeiformes* | 1 | *Anseriformes* | 9 | *Squamata* | 4 |  |  |
|  | *Carnivora* | 12 |  |  | *Cypriniformes* | 3 | *Apodiformes* | 2 | *Testudines* | 5 |  |  |
|  | *Chiroptera* | 1 |  |  | *Cyprinodontiformes* | 5 | *Bucerotiformes* | 2 |  |  |  |  |
|  | *Cingulata* | 1 |  |  | *Esociformes* | 1 | *Caprimulgiformes* | 2 |  |  |  |  |
|  | *Dasyuromorphia* | 1 |  |  | *Lepisosteiformes* | 1 | *Cariamiformes* | 2 |  |  |  |  |
|  | *Dermoptera* | 1 |  |  | *Perciformes* | 6 | *Cathartidae* | 1 |  |  |  |  |
|  | *Didelphimorphia* | 2 |  |  | *Pleuronectiformes* | 1 | *Charadriiformes* | 1 |  |  |  |  |
|  | *Lagomorpha* | 2 |  |  | *Salmoniformes* | 2 | *Coliiformes* | 2 |  |  |  |  |
|  | *Perissodactyla* | 6 |  |  | *Tetraodontiformes* | 2 | *Columbiformes* | 1 |  |  |  |  |
|  | *Primates* | 29 |  |  |  |  | *Coraciiformes* | 2 |  |  |  |  |
|  | *Rodentia* | 23 |  |  |  |  | *Cuculiformes* | 5 |  |  |  |  |
|  | *Scandentia* | 3 |  |  |  |  | *Euripygyformes* | 2 |  |  |  |  |
|  | *Soricomorpha* | 2 |  |  |  |  | *Falconiformes* | 2 |  |  |  |  |
|  | *Tubulidentata* | 1 |  |  |  |  | *Galliformes* | 3 |  |  |  |  |
|  |  |  |  |  |  |  | *Gaviiformes* | 2 |  |  |  |  |
|  |  |  |  |  |  |  | *Gruiformes* | 2 |  |  |  |  |
|  |  |  |  |  |  |  | *Leptosomiformes* | 2 |  |  |  |  |
|  |  |  |  |  |  |  | *Mesitornithiformes* | 2 |  |  |  |  |
|  |  |  |  |  |  |  | *Otidiformes* | 2 |  |  |  |  |
|  |  |  |  |  |  |  | *Passeriformes* | 14 |  |  |  |  |
|  |  |  |  |  |  |  | *Pelecaniformes* | 6 |  |  |  |  |
|  |  |  |  |  |  |  | *Phaethontiformes* | 2 |  |  |  |  |
|  |  |  |  |  |  |  | *Phoenicopteriformes* | 1 |  |  |  |  |
|  |  |  |  |  |  |  | *Piciformes* | 2 |  |  |  |  |
|  |  |  |  |  |  |  | *Podicipediformes* | 1 |  |  |  |  |
|  |  |  |  |  |  |  | *Procellariiformes* | 2 |  |  |  |  |
|  |  |  |  |  |  |  | *Psittaciformes* | 3 |  |  |  |  |
|  |  |  |  |  |  |  | *Pterocliformes* | 2 |  |  |  |  |
|  |  |  |  |  |  |  | *Sphenisciformes* | 4 |  |  |  |  |
|  |  |  |  |  |  |  | *Strigiformes* | 2 |  |  |  |  |
|  |  |  |  |  |  |  | *Struthioniformes* | 5 |  |  |  |  |
|  |  |  |  |  |  |  | *Suliformes* | 2 |  |  |  |  |
|  |  |  |  |  |  |  | *Tinamiformes* | 3 |  |  |  |  |
|  |  |  |  |  |  |  | *Trochiliformes* | 2 |  |  |  |  |
|  |  |  |  |  |  |  | *Trogoniformes* | 2 |  |  |  |  |
| Total |  | 97 |  | 1 |  | 25 |  | 103 |  | 12 |  | 2 |

**Supplementary table 2**. Sequences of several IAPP species with higher and lower aggregation propensity. Values of Aggrescan, PASTA, hydropathy, pI, µH and average hydrophobicity are showed, and in turn compared to human amylin sequence. *Hot spots* regions are identified in red.

| **Organism** | **Sequence** | **Aggrescan** | **PASTA** | **Hydropathy** | **pI** | **uH** | **Hidrophobicity** |
| --- | --- | --- | --- | --- | --- | --- | --- |
| *Pteropus alecto* | KCDTAIYAIQWLANFLVHSSNNFSVILSLTNVGSNTY | **20.9** | -12.882 | 0.36 | 7.2 | 0.16 | 0.25 |
| *Oryzias latipes* | KCNTATCVTQRLADFLVRSSNTIGTVYVPTNVGSATY | **7** | -6.288 | 0.1 | 9.0 | 0.21 | 0.07 |
| *Cyprinodon variegatus* | KCNTATCVTQRLADFLVRSSNTIGAVYVPTNVGSSTY | **6.6** | -5.707 | 0.1 | 9.0 | 0.20 | 0.07 |
| *Fundulus heteroclitusisoform x1* | KCNTATCVTQRLADFLVRSSNTIGTVYVPTNVGSSTY | **6.3** | -6.288 | 0.03 | 9.0 | 0.22 | 0.05 |
| *Xiphophorus maculatus* | KCNTATCVTQRLADFLVRSSNTIGTVYVPTNVGSSTY | **6.3** | -6.288 | 0.03 | 9.0 | 0.22 | 0.05 |
| *Austrofundulus limnaeus* | KCNTATCVTQRLADFLVRSSNTIGTVYVPTNVGSSTY | **6.3** | -6.288 | 0.03 | 9.0 | 0.22 | 0.05 |
| *Ochotona princeps* | KCNTITCATQRLANFLVHSSNNFGAIFSPVNLGSKSY | **5.2** | -4.341 | 0.04 | 9.7 | 0.26 | 0.10 |
| *Jaculus jaculus* | KCNTATCATQRLANFLVRSSSSLGVILPATNVGSNTY | **3.3** | -4.155 | 0.15 | 9.8 | 0.19 | 0.08 |
| *Danio* | KCNTATCVTQRLADFLIRSSNTIGTVYAPTNVGSATY | **3.2** | -4.252 | 0.04 | 9.0 | 0.22 | 0.07 |
| *Pterocles* | KCNTATCVTQRLADFLVRSSNNIGAIYSPTNVGSYTY | **3** | -3.568 | -0.12 | 8.9 | 0.23 | 0.03 |
| *Maylandia zebra* | KCNTATCVTQRLADFLVRSSNTIGTVYAPTNVGSATY | **2.6** | -4.284 | 0.03 | 9.0 | 0.22 | 0.06 |
| *Haplochromis burtoni* | KCNTATCVTQRLADFLVRSSNTIGTVYAPTNVGSATY | **2.6** | -4.284 | 0.03 | 9.0 | 0.22 | 0.06 |
| *Pundamilia nyererei* | KCNTATCVTQRLADFLVRSSNTIGTVYAPTNVGSATY | **2.6** | -4.284 | 0.03 | 9.0 | 0.22 | 0.06 |
| *Oreochromis niloticus* | KCNTATCVTQRLADFLVRSSNTIGTVYAPTNVGSATY | **2.6** | -4.284 | 0.03 | 9.0 | 0.22 | 0.06 |
| *Larimichthys crocea* | KCNTATCVTQRLADFLVRSSNTIGTVYAPTNVGSATY | **2.6** | -4.284 | 0.03 | 9.0 | 0.22 | 0.06 |
| ***Homo sapiens*** | **KCNTATCATQRLANFLVHSSNNFGAILSSTNVGSNTY** | **-5.6** | **-4.939** | **-0.1** | **9.1** | **0.20** | **0.06** |
| *Peromyscus maniculatus bairdii* | KCNTATCATQRLTNFLVRSSNNLGPVLPPTNVGSNTY | **-9.1** | -4.465 | -0.32 | 9.8 | 0.24 | -0.02 |
| *Bos_taurus* | KCGTATCETQRLANFLAPSSNKLGAIFSPTKMGSNTY | **-9.7** | -1.880 | -0.28 | 9.6 | 0.15 | 0.02 |
| *Erinaceus europaeus* | RCNTATCATQRLVNFLSRSSNNLGAILSPTDVGSNTY | **-9.9** | -4.384 | -0.21 | 9.1 | 0.24 | -0.04 |
| *Capra hircus* | KCGTATCATQRLANFLAPSGNKLGAVFSSTKMGSNTH | **-10.7** | -1.880 | -0.17 | 10.3 | 0.17 | 0.04 |
| *Cricetulus* | KCNTATCATQRLANFLVHSNNNLGPVLSPTNVGSNTY | **-10.8** | -3.884 | -0.27 | 9.1 | 0.20 | 0.03 |
| *Macaca fascicularis* | KCNTATCATQRLANFLVRSSNNFGTILSSTNVGSNTY | **-10.8** | -4.354 | -0.2 | 9.8 | 0.27 | -0.01 |
| *Octodon* | KCNTATCATQRLTNFLVRSSHNLGAALPPTKVGSNTY | **-11** | -4.465 | -0.29 | 10.2 | 0.21 | -0.03 |
| *Saimiri boliviensis boliviensis* | KCNTATCSMHRLADFLGRSGNNFGAILSPTNVGSNTY | **-11.3** | -1.880 | -0.26 | 9.1 | 0.27 | 0.02 |
| *Balearica regulorum gibbericeps* | KCNTATCSMHRLADFLGRSGNNFGAILSPTNVGSNTY | **-11.3** | -1.845 | -0.26 | 9.1 | 0.27 | 0.02 |
| *Sus scrofa* | KCNMATCATQHLANFLDRSRNNLGTIFSPTKVGSNTY | **-13.4** | -2.364 | -0.44 | 9.7 | 0.25 | -0.07 |
| *Ovis aries musimon* | KCGTATCATQRLANFLAPSGNKLGAVFSPRKMGSNTH | **-13.7** | -1.880 | -0.29 | 10.8 | 0.12 | -0.02 |
| *Odobenus rosmarus* | KCNTATRATQRLANFLVRSSNNLGAILSHTNVGSNTY | **-14.1** | -4.224 | -0.36 | 11.3 | 0.33 | -0.08 |
| *Bos mutus* | KCGTATCETQRLANFLAPSSNKLGAISSPTKMGSNTY | **-15.2** | -1.880 | -0.38 | 9.6 | 0.12 | -0.02 |
| *Bison bison bison* | KCGTATCETQRLANFLAPSSNKLGAISSPTKMGSNTY | **-15.2** | -1.880 | -0.38 | 9.6 | 0.12 | -0.02 |
| *Sorex araneus* | KCNTATCATQRLTNFLTRSSNNIGAIPPSTNVGSNTY | **-15.7** | -2.542 | -0.45 | 9.8 | 0.23 | -0.06 |

Hidrophobicity: hydrophobicity in kcal/mol.

**Supplementary figure 2.** Control experimentation on beta cells and hepatocytes under incubation with IAPP derived peptides. Tunicamycin (2 µg/mL) a broad characterized ER inducer, and PA (300 µM) were used as controls. Expression of ATF6α in beta cells (**A**), as well as CHOP (**B**) and IL-6 (**C**) in hepatocytes. Finally, on the same membranes and without stripping, and considering its high expression β-actin was used as a loading control.


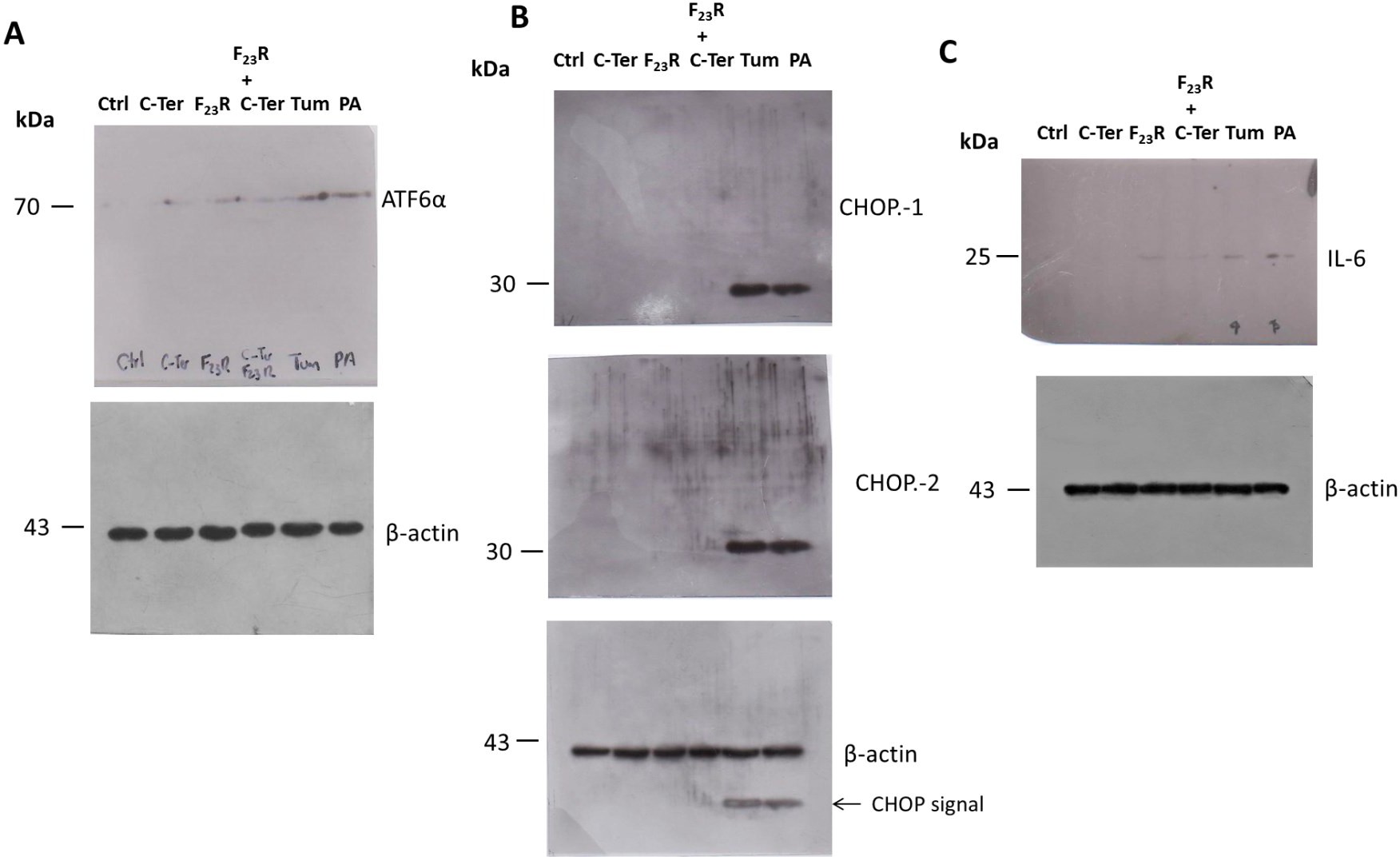

Supplement: Supplementary file 1 [file data_sheet_1.docx]
